# Effect of Plate Thickness on Residual Stress Distribution of GH3039 Superalloy Subjected to Laser Shock Peening

**DOI:** 10.3390/ma18153682

**Published:** 2025-08-05

**Authors:** Yandong Ma, Maozhong Ge, Yongkang Zhang

**Affiliations:** 1 School of Electromechanical Engineering, Guangdong University of Technology, Guangzhou 510006, China; zykseu@163.com; 2 Jiangsu Hengli Hydraulic Co., Ltd., Changzhou 213100, China; 3 School of Materials Engineering, Jiangsu University of Technology, Changzhou 213001, China

**Keywords:** laser shock peening, GH3039, J-C model, residual stress

## Abstract

To accurately assess the effect of different plate thicknesses on the residual stress field of laser shock peened GH3039 superalloy, residual stress measurements were performed on GH3039 alloy plates with thicknesses of 2 mm and 5 mm after laser shock peening (LSP) treatment. Both quasi-static and high strain rate mechanical tests of GH3039 were conducted, and the Johnson-Cook (J-C) constitutive equation for GH3039 alloy at specific strain rates was fitted based on the experimental results. To obtain the parameter *C* in the J-C constitutive equation of GH3039 alloy under ultra-high strain rates, a modified method was proposed based on LSP experiment and finite element simulation results. Using the modified GH3039 alloy J-C constitutive equation, numerical simulations and comparative analyses of the residual stress field of GH3039 alloy plates of different thicknesses under LSP were carried out using ABAQUS software. The simulated residual stress fields of laser-shocked GH3039 alloy plates of different thicknesses were in good agreement with the experimental measurements, indicating that the modified GH3039 alloy J-C constitutive equation can accurately predict the mechanical behavior of GH3039 alloy under ultra-high strain rates. Based on the modified GH3039 alloy J-C constitutive equation, the effect of different plate thicknesses on the residual stress distribution of laser-shocked GH3039 alloy was studied, along with the underlying mechanisms. The unique distribution characteristics of residual stresses in laser-shocked GH3039 plates with varying thicknesses are primarily attributed to differences in plate bending stiffness and the detrimental coupling effects of reflected tensile waves.

## 1. Introduction

GH3039, a solid-solution-strengthened nickel-based superalloy, is widely used in the manufacturing of high-temperature components for aviation engines due to its excellent high-temperature strength, oxidation resistance, and corrosion resistance. At room temperature, GH3039 alloy exhibits outstanding weldability and cold-forming capabilities, making it suitable for manufacturing lug components on the first-stage free turbine guide of a specific type of turbojet engine. Currently, shot peening technology is used by companies to improve the fatigue resistance of these lugs. However, fatigue cracking still occurs during operation under alternating loads, severely threatening the safety of aircraft operation. To prevent fatigue cracking without altering the material or structure of the lug, it is crucial to apply a new surface-strengthening process to enhance its fatigue resistance.

As a novel surface-strengthening technology, laser shock peening (LSP) has been widely used to improve the fatigue resistance of metal targets, utilizing ultra-high-pressure shock waves generated by nanosecond pulse lasers that strike the surface of the target. During propagation, most of the stress waves induce severe plastic deformation within the target, leading to the creation of fine grains and residual compressive stresses on both the surface and subsurface of the material, without damaging its structure, which effectively prevents the initiation and propagation of fatigue cracks [1,2]. Unlike shot peening, LSP can achieve a relatively smooth surface and a deeper deformation strengthening layer, significantly improving the fatigue resistance of the target material [3,4]. Recent experimental results from our research team have shown that the average fatigue life of GH3039 superalloy treated with LSP is more than two times that of untreated samples [5]. Additionally, flight tests conducted by our collaborating company have shown a significant improvement in the fatigue life of the GH3039 alloy lugs after LSP treatment. To expand the application of LSP to other parts made of GH3039 material, the company raised two questions: does the residual stress distribution remain consistent across different thicknesses of GH3039 parts under the same laser processing parameters? And, can the residual stress distribution of parts with varying thicknesses after LSP treatment be quickly predicted in future production processes? Given the widespread use of finite element analysis software in residual stress analysis, numerical simulations of the residual stress field in laser-shocked GH3039 alloy have become the preferred solution to address these questions. Recently, some researchers have studied LSP of superalloys using a numerical method. Ren et al. [6] investigated the microstructure evolution of LSP iron-based alloy GH2036 through experiment and simulation. Dislocation evolution during LSP GH2036 alloy was simulated by a dislocation dynamics method, and the dislocation density was measured. Kattoura et al. [7] proposed a method to determine the J-C equation parameters of LSP AA2024-T3, Ti-6Al-4V, and Inconel 718 alloys. The special feature of this method is that the strain-rate hardening coefficient *C* is calibrated and modified by comparing the Charpy impact indentation depth and the LSP-induced residual stress field from a numerical and experimental approach in turn. Fang et al. [8] found that for DD6 blades with [001] an orientation at 980 °C, three impacts can obtain the best compressive residual stress. In order to predict the LSP-induced residual stress field, the elastic–plastic constitutive model for nickel-based single crystal superalloy DD6 was constructed and ANSYS 12.1 was employed. Wu et al. [9] presented a method for calibrating the parameters of the J-C model of FGH4095 alloy. In the LSP numerical simulation, the strain-rate hardening coefficient *C* was randomly selected, and then modified according to the measured maximum compressive residual stress of LSP FGH4095. However, to the best of our knowledge, there are no related references to report the numerical simulation work about the LSP GH3039 superalloy, and there is also no constitutive model applicable to the GH3039 superalloy under ultra-high strain rates.

In this study, the residual stress distribution of laser-shocked GH3039 plates with varying thicknesses was predicted through numerical simulation, revealing the impact of plate thickness on the residual stress distribution. To determine the J-C constitutive equation for GH3039 alloy at a given strain rate, both room-temperature Hopkinson bar tests (SHPB) and uniaxial quasi-static tensile tests at different temperatures were performed. The J-C parameters *A*, *B*, *n*, *C*, and *m* were calibrated for GH3039 alloy using data fitting methods. Considering that the strain rate induced by LSP treatment can reach up to 10^6^ s^−1^ [7], the modified strain-rate hardening coefficient *C* was determined by comparing the measured and simulated surface residual stress values of LSP-treated GH3039 alloy. The accuracy of the J-C constitutive equation of GH3039 superalloy at ultra-high strain rate was further validated by comparing and analyzing the simulated and measured values of the residual stress field on the whole cross-section of GH3039 alloy plate after LSP. Using the validated J-C constitutive equation, ABAQUS software was employed to analyze the influence of plate thickness on the residual stress field of laser-shocked GH3039 alloy. To uncover the underlying mechanism, both a model for LSP-induced plate bending and an evolution model for the residual stress field of GH3039 plates with varying thicknesses were proposed. This not only enhances the application of LSP technology in aviation engine fields but also offers a novel approach for obtaining constitutive equations for materials under ultra-high strain rates.

## 2. Materials and Methods

### 2.1. Materials and the Specimen Preparation

**Figure 1:**
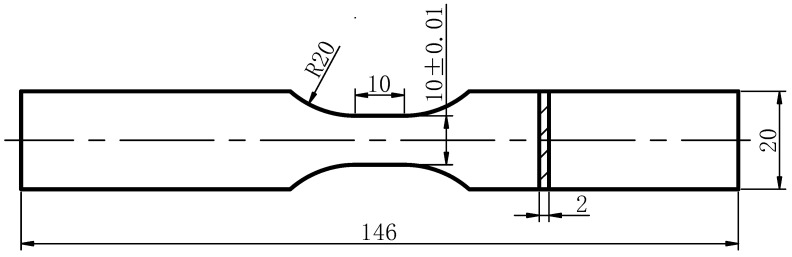
Dimensions of tensile sample at different temperatures.

The GH3039 superalloy ring-rolled billet, with a diameter of 350 mm and a height of 300 mm, was provided by Changzhou Lanxiang Machinery Co., Ltd. (Changzhou, Jiangsu Province, China). The chemical composition of the GH3039 alloy is listed in Table 1. Quasi-static tensile samples (as shown in Figure 1) were cut from the billet using a slow-wire electrical discharge machine (EDM). It is crucial to note that the length direction of the tensile samples was aligned with the rolling direction of the billet. Prior to tensile testing, the surfaces of all samples were sequentially polished with sandpaper, with grit sizes ranging from 600 to 2000 mesh. To achieve high strain rates, cylindrical samples with a diameter of 3 mm and a height of 2 mm were used for the separated Hopkinson bar test, with the height direction of the samples maintained parallel to the rolling direction. In order to ensure the parallelism and perpendicularity of the samples, the cylindrical billet was initially cut using the slow-wire EDM, after which both ends and the cylindrical surface of the samples were ground using a surface grinder and a centerless grinder, respectively, followed by polishing. Additionally, samples for residual stress measurement were processed by wire cutting, grinding, and polishing, and their dimensions were 24 mm × 24 mm in length and width, with heights of 2 mm and 5 mm. Before LSP treatment, all samples were ultrasonically degreased with acetone and allowed to air dry. The EDM was manufactured by Swiss GF Archimedel Group.(Geneva, Geneva Canton, Swiss); surface grinder and centerless grinder by Nantong Second Machine Tool Co., Ltd. (Nantong, Jiangsu Province, China).

### 2.2. LSP Procedure

LSP was performed using a Q-switched Nd: YAG high-power pulsed laser with a wavelength of 1064 nm, a pulse width of 20 ns, and a repetition rate of 10 Hz. To prevent thermal damage to the target surface, a 0.1 mm thick black tape was attached to the target, serving as an opaque overlayer. A 2 mm thick flowing water layer was applied on top of the black tape as a transparent overlayer, which helped to prolong the action time of the shock wave and enhance its peak pressure. The specific parameters used in the LSP process are as follows: a laser energy of 14 J, a laser beam with a diameter of 3.6 mm, and a spot overlap rate of 50%. The optimization method of laser energy is shown in Reference [10]. LSP processing equipment was manufactured by Xi’an Tianruida Laser Technology Co., Ltd. (Xi’an, Shanxi Province, China).

### 2.3. Residual Stress Measurement

**Figure 2:**
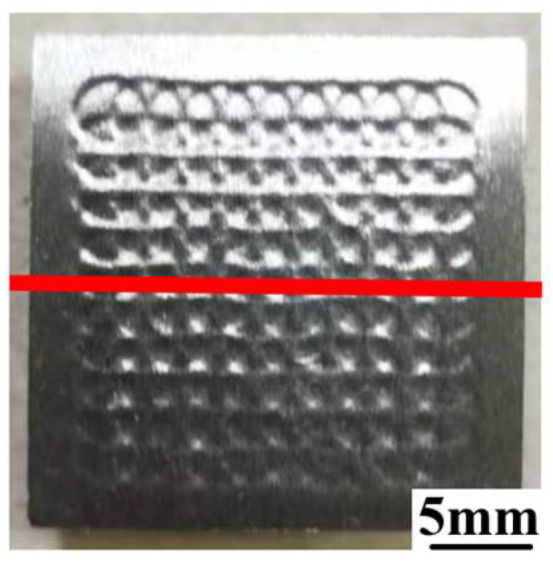
Measurement path (marked in red) of surface residual stress.

Residual stresses in the LSP-processed GH3039 were measured using the sin^2^ψ method with an XL-640 type X-ray stress measurer. An X-ray source operating at 6 mA and 22 kV was employed, and the (311) diffraction plane was selected for the analysis. The values of ψ were defined as 0°, 15.5°, 22.2°, 27.6°, 32.3°, 36.7°, 40.9°, and 45°, respectively. The stress constant was −289 MPa/(°). The scanning started at an angle of 163° and ended at 152°, with a scanning speed of 0.04°/s. The diameter of the X-ray beam was 3 mm. During the in-depth residual stress measurement, the subsurface material was removed through electro-polishing using a solution of 5% nitric acid and 95% alcohol (by volume), with a corrosion rate of approximately 0.3 μm/s per 1 cm^2^. To minimize measurement errors, measurements at each cross-section were repeated three times. The path for surface residual stress measurement (marked in red) is shown in Figure 2. During the measurement process, the distance between adjacent measuring points was maintained at 0.5 mm. X-ray stress measurer was manufactured by Handan Aiste Stress Technology Co., Ltd. (Handan, Hebei Province, China).

### 2.4. Selection of Constitutive Mode

It is well established that severe plastic deformation can be induced in materials by LSP at strain rates as high as 10^6^ s^−1^ [7]. At such ultra-high strain rates, the mechanical behavior of the material is significantly different from that observed under quasi-static conditions. Consequently, the selection of an appropriate dynamic constitutive model to accurately describe the transient stress–strain response of laser-shocked GH3039 alloy is essential. Under impact loading, the J-C model [11], the Steinberg model [12], and the Zerilli–Armstrong model [13] are commonly employed to characterize the transient stress–strain response of materials. Given that the J-C model is simple, physically meaningful, and relatively easy to parameterize, while having been successfully applied to characterize the dynamic properties of other metals under high strain rates, it has been selected in this study as the constitutive model for the GH3039 superalloy.

The J-C constitutive model is expressed as [11]:

(1) $$\sigma =(A+B\varepsilon^{n})(1+Cln{\dot{\varepsilon }}^{*})(1-T^{*m})$$

where *A* denotes the initial yield stress of the material at room temperature; *B*, *n*, *ε*, and *C* are defined as the strain-hardening coefficient, the strain-hardening exponent, the equivalent plastic strain and the strain-rate hardening coefficient, respectively; ${\dot{\varepsilon }}^{*}=\dot{\varepsilon }/{\dot{\varepsilon }}_{0}$ represents the dimensionless strain rate, with $\dot{\varepsilon }$ as the strain rate and ${\dot{\varepsilon }}_{0}$ the reference strain rate; *m* is thermal softening exponent; ${\;T}^{*}=\left(T-T_{ref}\right)/(T_{m}-T_{ref})\;$ is the homologous temperature with *T* being the current absolute temperature, $T_{m}$ the melting point of material and $T_{ref}$ as the reference temperature [11]. In this work, $T_{ref}$ is 298 K, ${\dot{\varepsilon }}_{0\;}$ is 0.001 s^−1^ and the melting point of GH3039 superalloy is 1670 K.

### 2.5. Quasi-Static Tensile Test at Different Temperatures

The parameters *A*, *B*, and *n* in the J-C constitutive model can be obtained through quasi-static tensile experiments conducted at room temperature, while the parameter *m* must be determined through quasi-static tensile tests at elevated temperatures. Uniaxial tensile tests were performed at various temperatures (298 K, 473 K, 573 K, 673 K, 773 K, and 873 K) using a Z250 electronic universal testing machine at a strain rate of 10^−3^ s^−1^. Prior to the high-temperature tensile tests, the samples were placed in a furnace and heated at a rate of 20 °C/min to the desired temperature, where they were held for 3 min to eliminate temperature gradients and ensure uniformity in sample temperature. During the tensile tests, all samples were stretched until fracture, with temperature and strain rate automatically adjusted throughout the process. Z250 electronic universal testing machine was manufactured by Shanghai Yuhan Machinery Co., Ltd. (Shanghai, China).

### 2.6. SHPB Test

To obtain parameter *C* in the J-C constitutive model, SHPB tests were conducted using the ZDSHPB-20 testing device. Since LSP is a cold working process rather than a hot working process, dynamic compression experiments were carried out at room temperature (298 K) under four different strain rates (4000 s^−1^, 6000 s^−1^, 8000 s^−1^, and 10,000 s^−1^). To minimize experimental errors, each set of tests was repeated three times. ZDSHPB-20 testing device was manufactured by Shandong Zongde Electromechanical Equipment Co., Ltd. (Jinan, Shandong Province, China).

### 2.7. LSP Numerical Simulation

**Figure 3:**
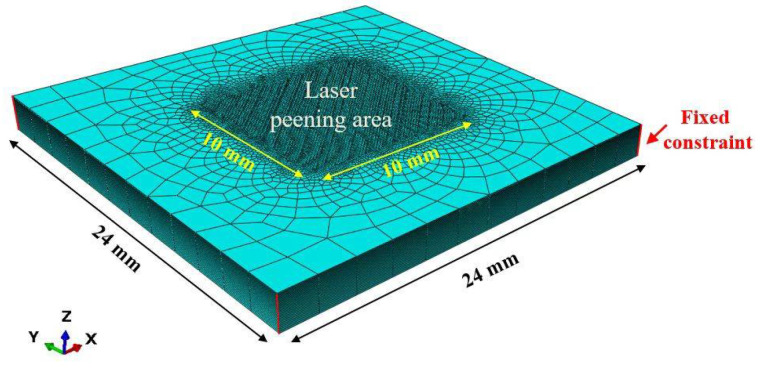
Schematic diagram of a model of a 2 mm thick specimen.

To predict the residual stress field generated in GH3039 alloy plates by LSP, a numerical analysis was performed using the commercial finite element analysis software ABAQUS with an “explicit–implicit” solution method. The length and width of the model were set to 24 mm × 24 mm respectively, and the fixed constraints are applied on the side of the model. Considering the varying thicknesses of actual GH3039 alloy parts, the model thicknesses were set to 2 mm, 3 mm, 4 mm, and 5 mm, respectively. Vasu et al. [14] believed that the laser shock region on the entire target can be replaced by selecting a typical region for modeling, which can save analysis time while ensuring analysis accuracy. The basis is that the influence range of a single laser shock is limited. To improve the computational efficiency, 9 spot-overlapping areas are selected as the representative areas of laser shock. The size of the mesh refinement area is 10 mm × 10 mm, the surface mesh size is set to 100 μm × 100 μm, and the depth mesh size is divided by a single bias strategy. In the multi-LSP simulation process, the interval between adjacent shock loads was set to 100,000 ns. The laser shock region of the 2 mm thick specimen contains 358316 C3D8R three-dimensional solid finite elements, as shown in Figure 3.

Under the constrained model, the peak pressure *P* (GPa) of the shock wave induced by LSP can be estimated using the following formula given by R. Fabbro [15]:

(2) $$P=0.01\times \sqrt{\frac{\alpha }{2\alpha +3}}\times \sqrt{Z}\times \sqrt{I_{0}}$$

where α denotes the coefficient of converting internal energy into thermal energy (with α = 0.2); *I*_0_ (GWcm^−2^) represents the laser energy intensity; and *Z* (gcm^−2^ s^–1^) is defined as the equivalent acoustic impedance, which can be expressed as follows [15]:

(3) $$\frac{2}{Z}=\frac{1}{Z_{1}}+\frac{1}{Z_{2}}$$

where *Z*_1_ and *Z*_2_ are defined as the acoustic impedance of constraint layer and target material, respectively.

The acoustic impedance of the target can be obtained according to the following equation:

(4) $$Z_{1}=\sqrt{\rho_{i}E_{i}}\times \sqrt{\frac{1-\upsilon_{i}}{\left(1+\upsilon_{i})(1-2\upsilon_{i}\right)}}$$

where *E_i_*, *ρ_i_*, and *υ_i_* represent the elastic modulus, the mass density, and Poisson’s ratio, respectively. In this study, GH3039 superalloy was employed as the target material, with an elastic modulus of 196 GPa, a mass density of 8.3 g/cm^3^, and a Poisson’s ratio of 0.3. Therefore, the acoustic impedance of GH3039 is 4.68 × 10^6^ g/(cm^2^ s). Additionally, the acoustic impedance of flowing water, used as the constraint layer, is 1.65 × 10^5^ g/(cm^2^ s). By substituting these two parameters into Equation (3), the equivalent acoustic impedance is determined to be 3.188 × 10^5^ g/(cm^2^ s). The laser energy density can be estimated according to the following equation:

(5) $$I_{0}=\frac{4E}{\pi d^{2}\tau }$$

where *E*, *d,* and *τ* represent the laser energy, the diameter of laser spot, and laser pulse duration, respectively. By substituting the above parameters into Equations (2)–(5), the peak pressure of the LSP-induced impact wave is calculated to be approximately 3.59 GPa.

**Figure 4:**
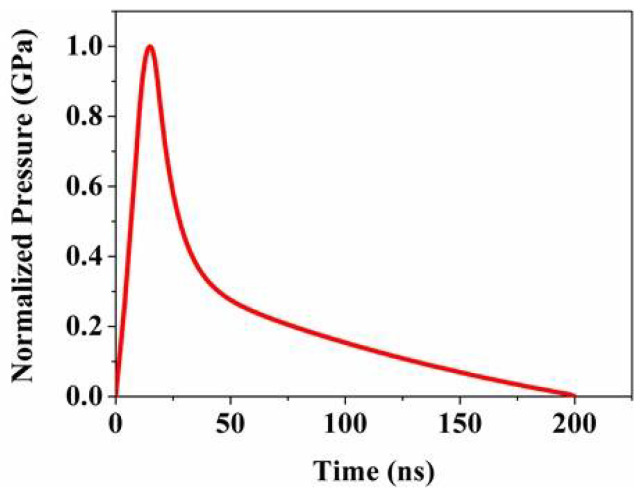
Temporal profile of normalized pressure pulse.

**Figure 5:**
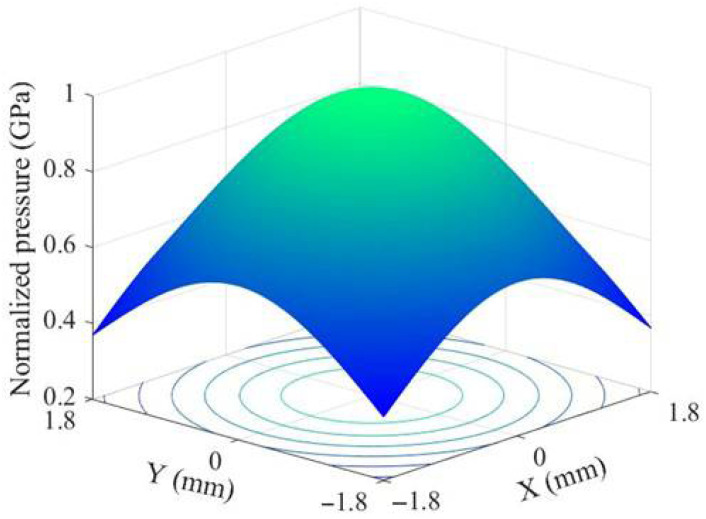
Spatial distribution of normalized impact wave pressure.

In the current study, the laser pulse duration is 20 nanoseconds, and the duration of the laser-induced shock wave is approximately 2–3 times that of the laser pulse duration [15]. The normalized pressure–time distribution curve of the LSP-induced shock wave is shown in Figure 4. Additionally, the laser pulse energy used in the experiment follows a Gaussian distribution. Since the spatial distribution of the shock wave pressure closely resembles that of the laser beam energy distribution, the time–space distribution formula for the normalized shock wave pressure is as follows:

(6) $$P(r,t)=P(t)\times exp(-\frac{r^{2}}{2R^{2}})$$

where *R* represents the laser spot radius, *r* is the distance from the center of the spot, and *P*(*t*) denotes the normalized pressure of the shock wave as a function of time. The spatial distribution of the normalized pressure pulse is shown in Figure 5.

## 3. Results and Discussion

### 3.1. Establishment and Validation of J-C Constitutive Equation of LSP-Processed GH3039

#### 3.1.1. Establishment of J-C Constitutive Equation of GH3039 at Strain Rate of 10^4^ s^−1^

When $\dot{\varepsilon }={\dot{\varepsilon }}_{0}$ and $T=T_{ref}$ are considered, both the second and third terms on the right-hand side of Equation (1) equal 1. Therefore, Equation (1) can be simplified to the following formula:

(7) $$\sigma -A=B\varepsilon^{n}$$

Taking the natural logarithm of both sides of Equation (7), the following equation can be obtained:

(8) ln(σ−A)=lnB+nlnε

**Figure 6:**
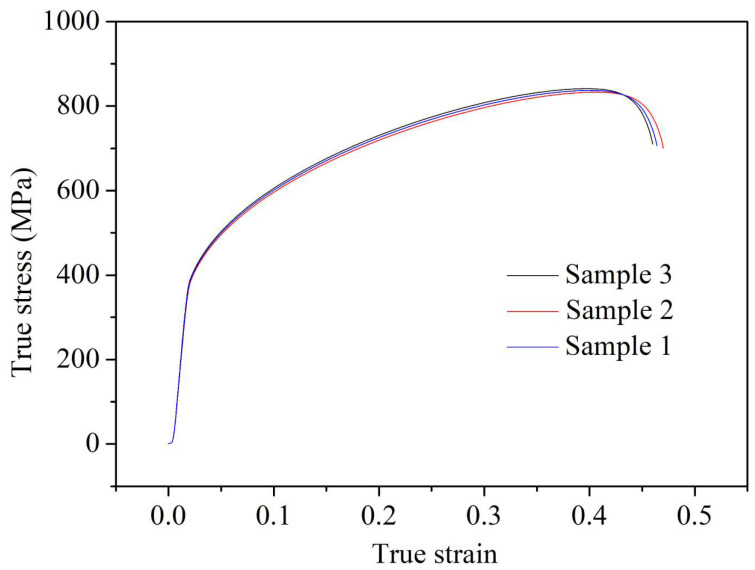
True stress–strain curves of GH3039 specimens during quasi-static tension at room temperature with strain rate of 0.001 s^−1^.

The true stress–strain curve of GH3039 superalloy during the quasi-static tensile process at room temperature and a strain rate of 0.001 s^−1^ is shown in Figure 6. It can be observed from Figure 6 that a distinct yield phase is not exhibited by the GH3039 alloy during tensile deformation. Therefore, the stress value corresponding to a 0.2% plastic strain is used as the nominal yield strength, which is A = 399.8 MPa.

**Figure 7:**
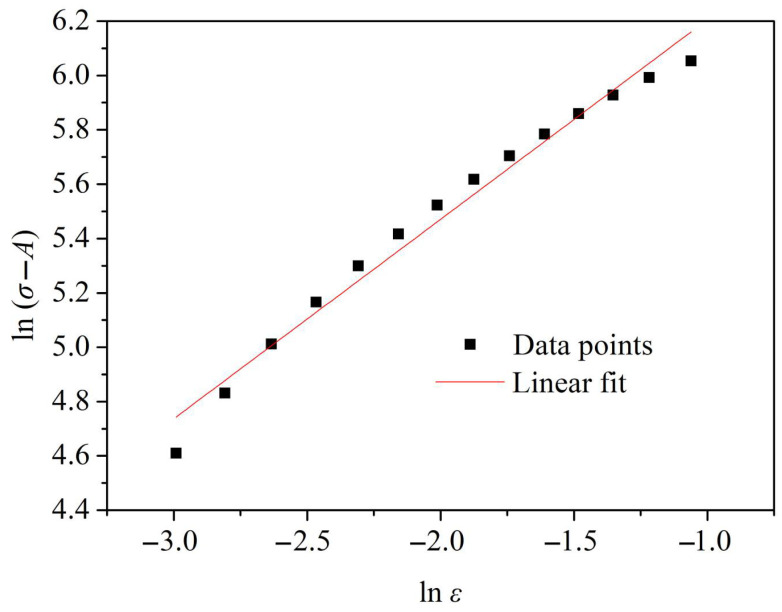
Fitting diagram of *n* and *B*.

By substituting the average stress of three samples at the same strain into Equation (8), the fitting line for the parameters $ln\left(\sigma -A\right)$ and lnε is obtained, as shown in Figure 7. A correlation coefficient of 0.99 is reached for this fitting line, indicating an excellent fit. From the slope and intercept of the fitting line in Figure 7, it is determined that *n* = 0.734 and *B* = 1031.9 MPa.

Similarly, when $\dot{\varepsilon }={\dot{\varepsilon }}_{0}$, the second term on the right-hand side of Equation (1) equals 1. Therefore, Equation (1) can be rewritten as:

(9) $$1-\sigma /\left(A+B\varepsilon^{n}\right)=T^{*m}$$

Taking the natural logarithm of Equation (9), Equation (9) can be gained as follows:

(10) $$ln[1-\sigma /\left(A+B\varepsilon^{n}\right)]=mlnT^{*}$$

**Figure 8:**
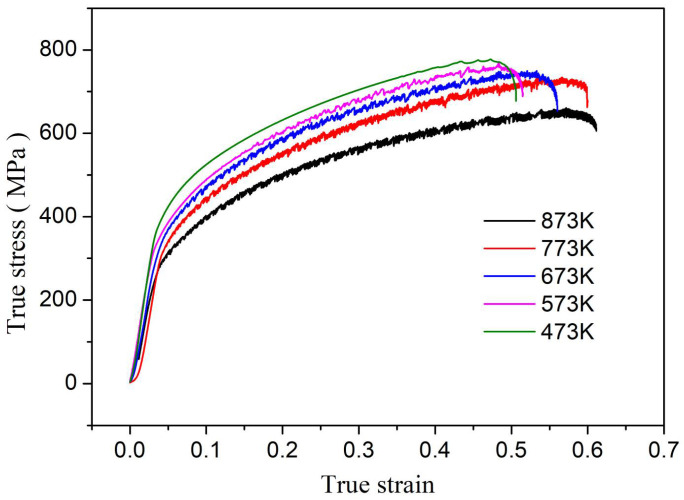
True stress–strain curves of GH3039 specimens during quasi-static tension at different temperatures with strain rate of 0.001 s^−1^.

The true stress–strain curves of GH3039 alloy during quasi-static tensile testing at the same strain rate but at different temperatures are shown in Figure 8. It can be observed from Figure 8 that a significant temperature-softening effect is exhibited by GH3039 superalloy. Specifically, under the same strain rate conditions, the true stress during the plastic deformation phase decreases as the temperature increases.

**Figure 9:**
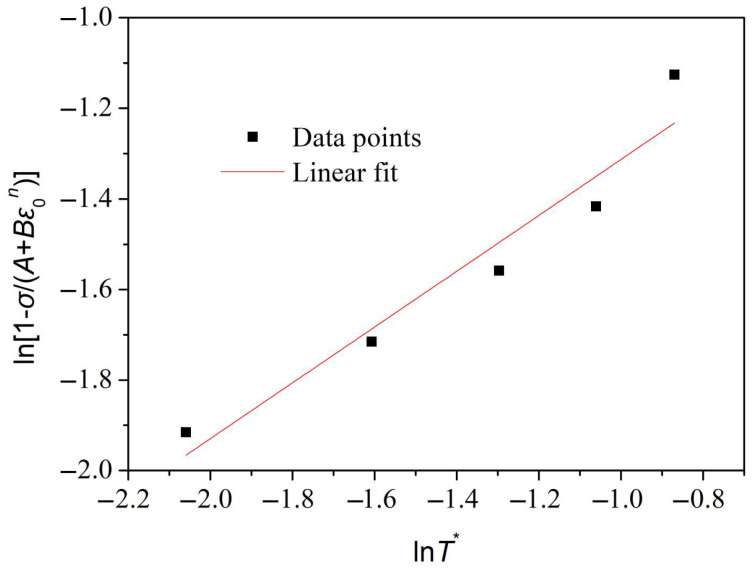
Fitting diagram of $ln[1-\sigma /\left(A+B\varepsilon^{n}\right)]$ and $lnT^{*}$.

Equation (10) indicates that $ln[1-\sigma /\left(A+B\varepsilon^{n}\right)]$ is proportional to $lnT^{*}$. By substituting the stress values corresponding to selected strains at five different experimental temperatures into Equation (10), the fitting line between $ln[1-\sigma /\left(A+B\varepsilon^{n}\right)]$ and $lnT^{*}$ is obtained, as shown in Figure 9. A correlation coefficient of 0.97 is reached for this fitting line, indicating an excellent fit. From the slope of the fitted line, the thermal softening index *m* is calculated to be 0.62.

Additionally, when $T=T_{ref}$ are considered, the third term on the right-hand side of Equation (1) equals 1. Therefore, Equation (1) can be rewritten as

(11) $$\sigma /(A+B\varepsilon^{n})-1=Cln{\dot{\varepsilon }}^{*}$$

**Figure 10:**
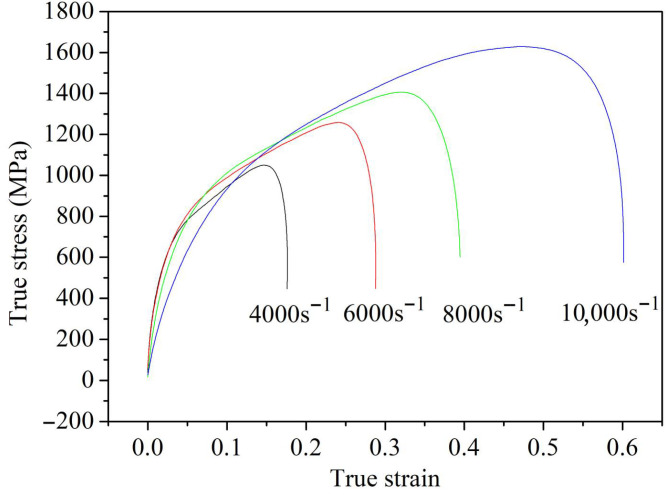
True stress–strain curves of GH3039 specimens under dynamic compression at room temperature and various strain rates.

The true stress–strain curves of GH3039 alloy during dynamic compression at room temperature and different strain rates are shown in Figure 10. It can be observed from Figure 10 that as the strain rate increases, the plastic deformation phase of GH3039 alloy is significantly prolonged. Moreover, a strain-rate-strengthening effect is exhibited by GH3039 superalloy at room temperature, where the true stress increases with increasing strain rate due to the gradual increase in dislocation density, leading to dislocation entanglement, intersection, and other obstacles. These factors result in increased dislocation motion resistance and deformation resistance, thereby generating the strain-rate-strengthening effect [16].

**Figure 11:**
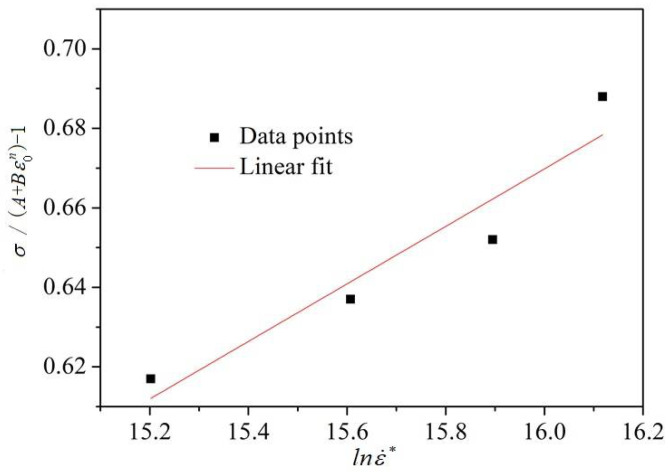
Fitting diagram of $\sigma /(A+B\varepsilon^{n})-1$ and $ln{\dot{\varepsilon }}^{*}$.

Similarly, Equation (11) indicates a linear relationship between $\sigma /(A+B\varepsilon^{n})-1$ and $ln{\dot{\varepsilon }}^{*}$. By substituting the stress values corresponding to selected strains at four different strain rates into Equation (11), the fitting line between $\sigma /(A+B\varepsilon^{n})-1$ and $ln{\dot{\varepsilon }}^{*}$ is obtained, as shown in Figure 11. The correlation coefficient of this fitting line reaches 0.95, indicating a good fit. From the slope of the fitted line, the strain-rate-strengthening coefficient is calculated to be 0.072.

Based on the above experimental and fitting results, the J-C constitutive equation for GH3039 superalloy at a strain rate of 10^4^ s^−1^ is derived as follows:

(12) $$\sigma =(399.8+1031.9\varepsilon^{0.734})(1+0.072ln{\dot{\varepsilon }}^{*})(1-T^{*0.62})$$

#### 3.1.2. Establishment of J-C Constitutive Equation of GH3039 at Ultra-High Strain Rate

Since the strain rate achievable in separated Hopkinson bar (SHPB) experiments is limited to 10^4^ s^−1^, while the strain rate induced in the target material by LSP reaches up to 10^6^ s^−1^, this study proposes a modified method based on LSP experiment and finite element simulation results to obtain the parameter *C* at ultra-high strain rates.

**Figure 12:**
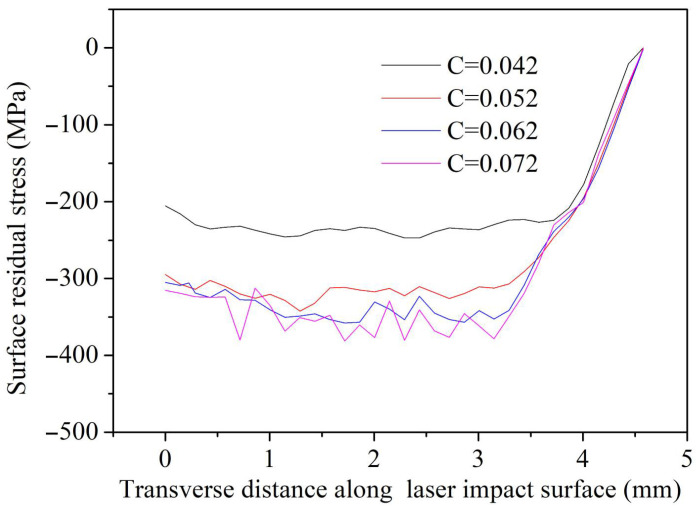
Surface residual stress distributions of laser-treated GH3039 obtained from simulation under different parameter *C*s.

**Figure 13:**
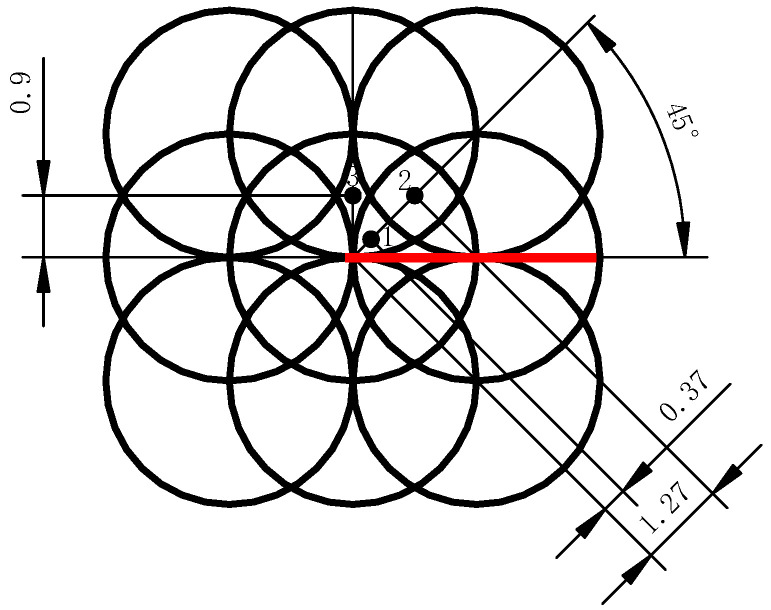
Extraction path of surface residual stress distribution (marked in red) and extraction position of through-thickness residual stress distribution (marked with numbers).

Figure 12 presents the surface residual stress distribution of 2 mm thick GH3039 alloy under different parameter *C*s obtained through numerical simulation. The residual stress extraction path on the surface (marked in red) and the residual stress extraction region in the depth direction (marked with numbers) is shown in Figure 13. Figure 12 indicates that the distribution trends of surface residual stress are essentially consistent across different values of parameter *C*. In the LSP region, the residual compressive stress is relatively high, but as one moves away from the laser impact zone, the residual compressive stress rapidly decreases and approaches zero. Furthermore, after multiple LSP treatments, the surface residual stress distribution along the central path of the impacted area exhibits a characteristic “sine wave” pattern, which becomes more pronounced as parameter *C* increases. Based on the surface residual stress values extracted from the LSP area, the average surface residual stresses are −232.7 MPa, −317.5 MPa, −328.6 MPa, and −338.0 MPa, corresponding to parameter *C* values of 0.042, 0.052, 0.062, and 0.072, respectively. Therefore, it can be inferred that the surface residual stress induced by LSP increases with the value of parameter *C*. Table 2 presents the measured surface residual stresses at different positions of the laser-shocked GH3039 alloy. From Table 2, the surface residual stress changes with the measurement position, which is mainly due to the uneven spatial distribution of the shock wave energy induced by LSP. Additionally, the average surface residual stress measured for the laser-shocked GH3039 alloy is −319.1 MPa. By comparing the simulated values with the measured values, it is found that when parameter *C* = 0.052, the average surface residual compressive stress of GH3039 alloy is closest to the experimental value, with an error of only 0.51%. Therefore, the modified J-C constitutive equation for GH3039 superalloy is as follows:

(13) $$\sigma =(399.8+1031.9\varepsilon^{0.734})(1+0.052ln{\dot{\varepsilon }}^{*})(1-T^{*0.62})$$

#### 3.1.3. Validation of J-C Constitutive Equation of GH3039 at Ultra-High Strain Rate

**Figure 14:**
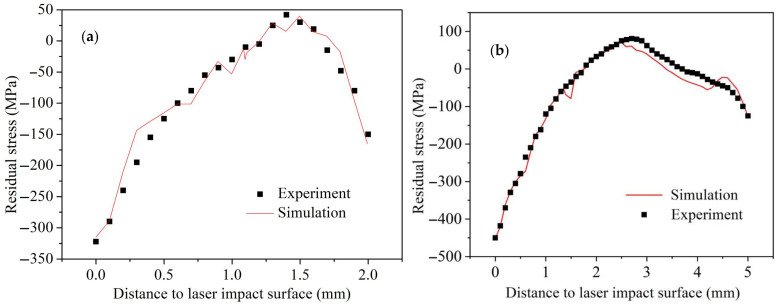
Experimental and simulated in-depth residual stress distributions of 2 mm (**a**) and 5 mm (**b**) GH3039 plate by one-sided LSP.

To further validate the accuracy of the J-C constitutive equation for GH3039 superalloy at ultra-high strain rates, the residual stress distribution across the entire cross-section of 2 mm and 5 mm thick laser-shocked GH3039 alloy plate was analyzed by comparing experimental measurements with numerical simulation results, as shown in Figure 14. It is important to note that the residual stress along the depth direction is the average value of the three different extraction regions shown in Figure 13. From Figure 14, it can be demonstrated that the trends in residual stress distribution along the thickness direction for both experimental measurements and simulations are essentially the same. The measured residual stress values at each depth are very close to the simulated values, and the depth that the measured and simulated residual compressive stress can reach is also basically close. This confirms that the J-C constitutive equation for GH3039 superalloy at ultra-high strain rates can accurately predict the residual stress field induced by LSP on GH3039 alloy.

Currently, the majority of the literature indicates that, following one-sided LSP, the final stress distribution across the entire cross-section of the target material consists of compressive residual stress on the laser-shocked side and tensile residual stress on the non-shocked side, thereby maintaining the internal stress equilibrium of the material [17]. However, in this study, the residual stress field across the entire cross-section of the 2 mm thick GH3039 plate along the laser shock direction consists of three parts: compressive residual stress on both the laser-peened side and the non-peened side, with tensile residual stress sandwiched between them. Additionally, previous studies have successfully produced samples with a uniform residual compressive stress field across the entire cross-section using one-sided LSP. Ocaña et al. [18] reported that, after one-sided LSP, a residual compressive stress field was induced across the entire cross-section of 2 mm thick Al2024-T351 aluminum alloy plates. However, Meng et al. [19] pointed out that, for the same thickness of Al2024-T351 plates, after one-sided LSP treatment, the depth of the residual compressive stress layer on the laser-shocked side was only 0.712 mm. The differences in the residual stress field for plates of the same thickness and material after one-sided LSP are primarily attributed to variations in laser power density.

### 3.2. Residual Stress Distributions as a Function of Plate Thickness After One-Sided LSP

**Figure 15:**
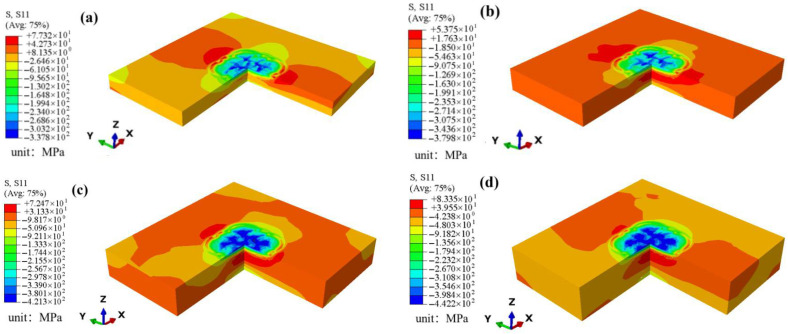
Residual stress contours of the 2 mm (**a**), 3 mm (**b**), 4 mm (**c**) and 5 mm (**d**) GH3039 plates subjected to one-sided LSP.

Based on the validated model, the residual stress field induced in GH3039 plates of different thicknesses after one-sided LSP can be easily predicted. Figure 15 shows the distribution of the residual stress contours in GH3039 alloy plates after one-sided LSP, with the residual stress distribution varying with plate thickness. In Figure 15, panels (a), (b), (c), and (d) correspond to plate thicknesses of 2 mm, 3 mm, 4 mm, and 5 mm, respectively. Different residual stresses are represented by different colors. It can be observed that, under the same processing parameters, the surface and bottom layers of the laser-shocked GH3039 alloy plates exhibit compressive residual stress, while tensile residual stress is present in the middle region of the plate. Additionally, the thickness of the tensile stress layer increases with the increase in plate thickness.

**Figure 16:**
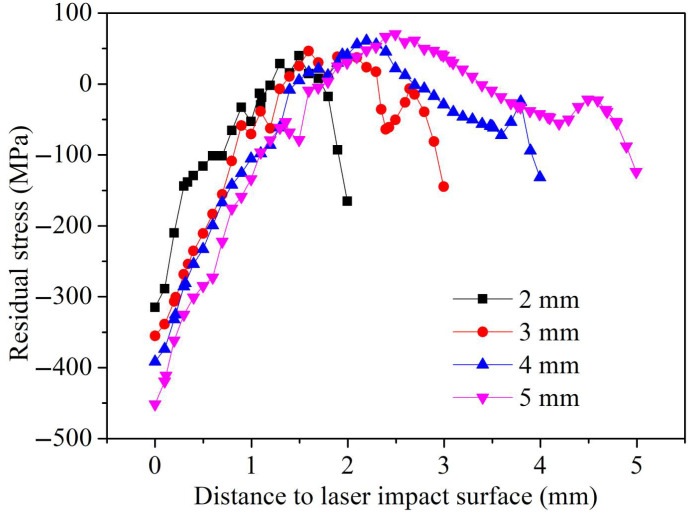
In-depth residual stress distributions in GH3039 sheets of different thicknesses treated by one-sided LSP treatment.

The variation in the residual stress distribution within GH3039 alloy plates subjected to LSP with different plate thicknesses under the same processing parameters is illustrated in Figure 16. It should be noted that the residual stress values along the depth direction are the average results obtained from measurements in three different sampling regions, as shown in Figure 13. The simulated residual stress values for the laser-shocked GH3039 alloy plates are found to be in close agreement with the measured values. From Figure 16, it can be observed that for the 2 mm, 3 mm, 4 mm, and 5 mm thick laser-shocked GH3039 alloy plates, the highest residual compressive stresses on the laser-shocked surface are −314.9 MPa, −355.2 MPa, −391.4 MPa, and −451.6 MPa, respectively. The residual compressive stresses on the back surface of the plates are −165.2 MPa, −144.7 MPa, −131.5 MPa, and −123.8 MPa, respectively. It can be concluded that as the plate thickness increases, the residual compressive stress on the laser-shocked surface increases, while the residual compressive stress on the back surface decreases. Furthermore, as the thickness increases from 2 mm to 5 mm, the depth of the residual compressive stress layer on the laser-shocked surface increases from approximately 1.2 mm to 1.7 mm, and the residual compressive stress at the same depth also increases correspondingly. Additionally, the residual compressive stress on the laser-shocked side of the GH3039 alloy plate is observed to be significantly greater than that on the back surface. In summary, significant differences in both the magnitude and distribution of residual stress are exhibited by the laser-shocked GH3039 alloy plates with different thicknesses.

### 3.3. Bending Deformation Degree as a Function of Plate Thickness After One-Sided LSP

**Figure 17:**
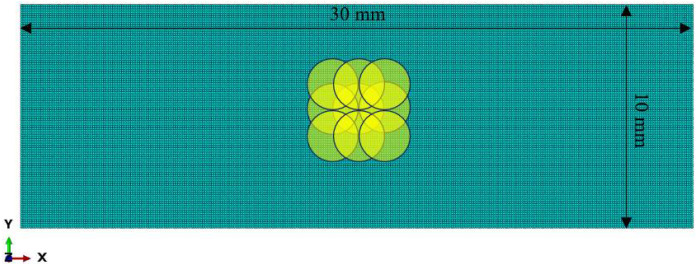
Schematic diagram of a model of bending deformation analysis.

**Figure 18:**
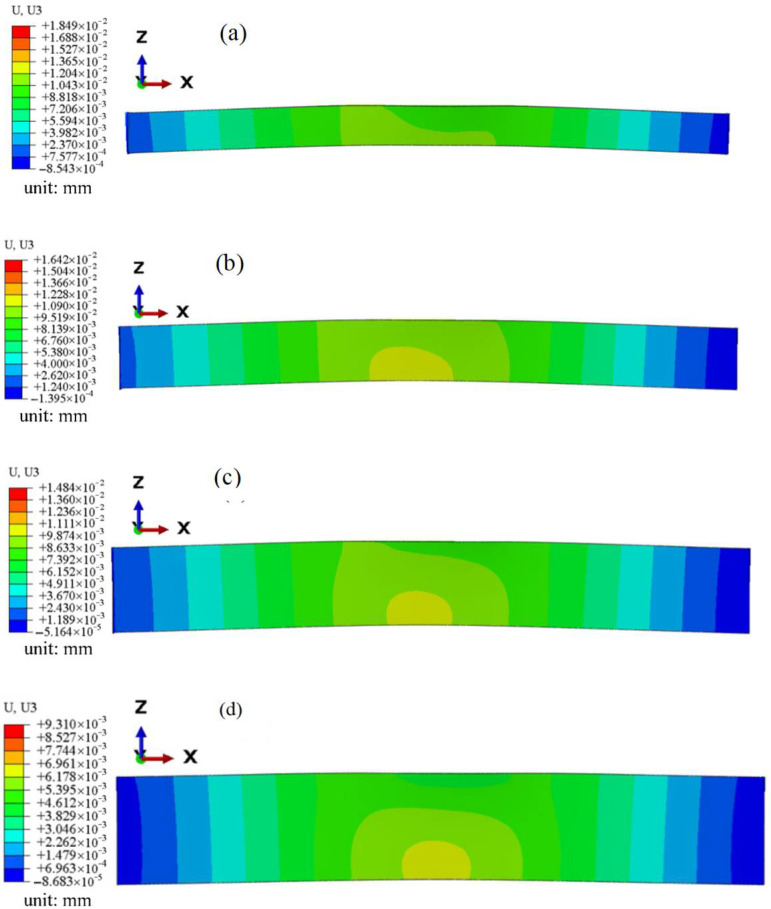
Displacement contours of the 2 mm (**a**), 3 mm (**b**), 4 mm (**c**), and 5 mm (**d**) GH3039 plates treated by one-sided LSP.

To predict the deformation of GH3039 alloy plates induced by LSP, finite element analysis was conducted using an “explicit–implicit” solution method. The model dimensions were 30 mm × 10 mm, with a surface mesh size of 100 μm. The reason why the cuboid model is selected is to more significantly reflect the deformation degree of the GH3039 sheet after bending. A single offset strategy was employed to divide the depth mesh. All four edges of the model were fully constrained. In this study, nine laser spots applied to the central region of the model were used to predict the deformation patterns of laser-shocked GH3039 alloy plates with different thicknesses, as shown in Figure 17. The GH3039 constitutive model and the laser-induced shock wave-loading model remained unchanged. Figure 18 presents the displacement contours of GH3039 alloy plates with different thicknesses after one-sided LSP, visually illustrating the degree of bending deformation of the plates. The degree of bending deformation (also referred to as the arc height value) is represented by the displacement along the Z-axis at the midpoint of the plate’s length direction. From Figure 18, it can be observed that under the same simulation conditions, the arc heights for the 2 mm, 3 mm, 4 mm, and 5 mm thick laser-shocked GH3039 alloy plates are 0.0185 mm, 0.0164 mm, 0.0148 mm, and 0.0093 mm, respectively. Therefore, it can be inferred that, under the same processing conditions, as the plate thickness increases, the degree of bending deformation in the one-sided laser-shocked GH3039 plates decreases due to the lower stiffness of thinner plates and the higher stiffness of thicker plates.

### 3.4. Influence Mechanism of Plate Thickness on the Residual Stress Field of LSP-Processed GH3039

**Figure 19:**
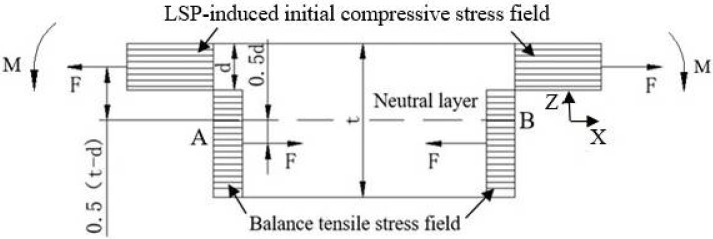
Schematic illustration of bending moment generated by single-side laser shock in GH3039 plate before bending deformation.

There is an inherent stress state inside any untreated material. Regardless of the initial stress state, the stress system inside the whole material is self-balanced without external force. This means that the stress at any point inside the material satisfies the equilibrium state. During the LSP process, the initial compressive stress layer induced by the laser shock disrupts the original stress equilibrium within the target material. To restore the internal stress equilibrium, a corresponding tensile stress layer is automatically generated in the matrix of the material when the initial compressive stress layer is formed. For the sake of simplification, it is assumed that, under the same laser parameters, the weakening effect of the reflected tensile wave is negligible. Therefore, the initial compressive stress field in samples of different thicknesses is approximately the same. It is also assumed that both the laser-induced initial compressive stress field and the resulting tensile stress field are uniformly distributed. Figure 19 illustrates the cause of the bending in GH3039 plates after one-sided LSP. The shock wave generated by the pulsed laser acts perpendicularly on the surface of the plate and propagates inward. Plastic deformation of the plate surface occurs only when the peak pressure of the shock wave exceeds the dynamic yield strength of the plate. The matrix, however, undergoes elastic deformation. The plastic deformation causes elongation of the metal surface material, leading to an increase in the surface area of the laser shock-peened region. Due to the irreversible plastic deformation on the plate surface, residual compressive stress is generated within the LSP-affected layer, which impedes the elastic recovery of the matrix. According to the principle of action and reaction forces, a corresponding tensile stress is generated in the matrix. Due to the asymmetric structure between the matrix and the LSP-affected layer, when the affected layer elongates, the sample experiences an additional bending moment. To achieve a state of self-balance, the plate bends downward under the influence of this bending moment. However, the downward bending of the GH3039 plate fundamentally alters the initial stress field induced by LSP. In Figure 19, the initial compressive stress field (with a depth of *d*) and the corresponding tensile stress field (with a depth of *t-d*) have equivalent magnitudes of force, both represented by *F*. The points of action of these forces are located at a distance of 0.5 (*t-d*) and 0.5*d* from the neutral layer on each side. Therefore, the resultant moment *M* of the left pair of forces relative to point A is given by

(14) M=Ft/2

where *t* is the thickness of the GH3039 plate. The direction of the resultant *M* is shown in Figure 19. Equation (14) indicates that the resultant moment *M* is proportional to the plate thickness *t* and the equivalent force *F*, with the equivalent force depending on the magnitude and distribution depth of the initial compressive stress induced by LSP. Similarly, a reverse bending moment exists on the right side. Under the action of these two moments, the plate will bend downward, forming a bulging structure.

The stress in the cross-section of the plate caused by the bending moment *M* can be expressed as

(15) $$\sigma =\frac{Mz}{I_{y}}=\frac{Mz}{bt^{3}/12}$$

where *I_y_* represents the second axial moment of area, which reflects the bending stiffness of the plate; *b* and *t* denote the width and thickness of plate, respectively; *z* represents the distance from the neutral axis of the cross-section. In this work, *b* is constant, while *t* is a variable. Equation (15) indicates that the bending stress at *z* is inversely proportional to the cube of the plate thickness and proportional to the bending moment. Therefore, under the same bending moment *M*, the bending stress at position *z* is greater in the thin plate than in the thick plate. When z=t/2, $\sigma_{max}=6M/bt^{2}=3F/bt$ can be achieved, which means that under the same equivalent force *F*, the maximum bending stress on the cross-section of the thin plate is greater than the maximum bending stress on the cross-section of the thick plate.

**Figure 20:**
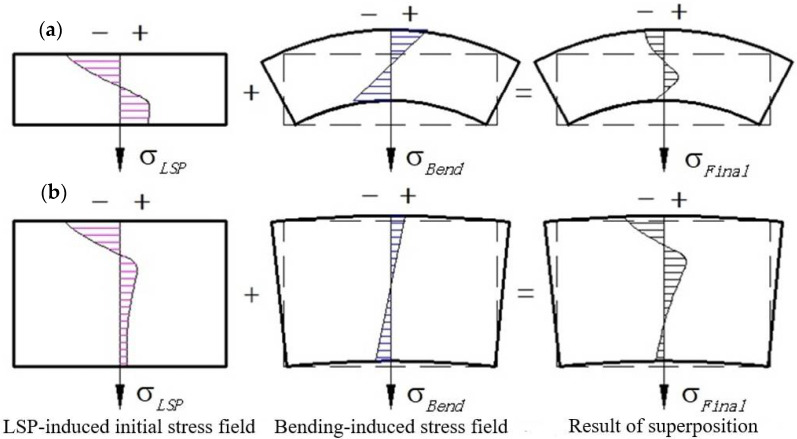
A schematic of influence of plate thickness on evolution of residual stress field in LSP GH3039 plate: thin plate (**a**) and thick plate (**b**).

Essentially, the final residual stress distribution in the laser-shocked GH3039 alloy plate is the linear superposition of the initial stress field induced by LSP in the thickness direction and the stress field caused by the plate bending, as shown in Figure 20. Due to the cancellation of tensile and compressive stress during superposition, the initial stress field induced by LSP in the GH3039 plate undergoes fundamental changes. It is evident that the greater the bending tensile stress, the higher the degree of cancellation of the original compressive stress on the laser-shocked surface. Compared to thin plates, thick plates have greater bending stiffness and resistance to bending, resulting in smaller bending deformation and bending stress under the same bending moment. Consequently, the stress compensation effect on the laser-treated surface is weaker. As a result, as the plate thickness increases, both the magnitude and the distribution depth of the residual compressive stress on the laser-treated surface of the GH3039 alloy plate gradually increase.

Moreover, the weakening effect of the reflected wave on the residual stress field increases as the plate thickness decreases. Under the same laser processing conditions, the peak pressure of the shock wave acting on the surface of GH3039 alloy plates of different thicknesses remains constant. When the shock wave generated by LSP reaches the target surface, both reflection and transmission phenomena occur. The transmitted wave consists of elastic compression waves and plastic compression waves, with the wave speed of the elastic compression wave being higher than that of the plastic compression wave. For thin plates, the attenuation of the elastic compression wave in the thickness direction is minimal, allowing it to quickly propagate to the back surface of the target where it is strongly reflected as an elastic tensile wave. As the plastic compression wave interacts with the reflected tensile wave, its amplitude is significantly reduced, thus weakening the residual compressive stress field in the laser-shocked region. As plate thickness increases, the propagation distance of the elastic compression wave in the thickness direction also increases, leading to greater attenuation. Consequently, the intensity of the elastic compression wave reaching the back surface of the target becomes small or negligible, resulting in a reduction or elimination of the corresponding reflected tensile wave intensity. When smaller reflected tensile waves interact with the initial plastic compression wave within the material, the compressive residual stress on the laser-treated surface increases in both magnitude and distribution depth due to the weakening of the harmful coupling effect between the two waves.

In conclusion, the distribution of the residual stress field in GH3039 superalloy under varying laser processing parameters can be efficiently determined with the assistance of the GH3039 J-C constitutive model and numerical simulations. Should the resulting stress field fail to meet the service requirements for aerospace components, adjustments to the laser processing parameters can be made, and the simulation process can be repeated until the desired outcome is achieved. This approach not only eliminates the need for the complex and time-consuming residual stress measurement process but also enables computational control over the effects of LSP.

## 4. Conclusions

Through the experimental characterization of laser-shocked GH3039 superalloy, the creation of the J-C constitutive equation, and finite element simulation analysis, the following conclusions can be drawn:

(1) Based on the results of quasi-static tensile tests and separated Hopkinson bar tests, the J-C constitutive equation for GH3039 under a given strain rate was derived through data fitting.
(2) By comparing finite element simulations with LSP experimental results, the strain-rate coefficient *C* in the GH3039 J-C constitutive equation was modified. A constitutive equation for GH3039 at ultra-high strain rates is $\sigma =(399.8+1031.9\varepsilon^{0.734})(1+0.052ln{\dot{\varepsilon }}^{*})(1-T^{*0.62})$.
(3) The final residual stress distribution in the laser-shocked GH3039 alloy plate is the linear superposition of the initial stress field induced by LSP in the thickness direction and the stress field caused by the plate bending.
(4) The residual stress field in GH3039 alloy plates is significantly influenced by plate thickness under the same LSP conditions. As the plate thickness increases, both the magnitude and the distribution depth of the residual compressive stress on the laser-treated surface of the GH3039 plates exhibit an increasing trend. This is primarily attributed to the higher bending stiffness of thicker plates, which reduces the harmful coupling effect caused by the reflected tensile wave.
(5) With the help of the GH3039 J-C constitutive model and numerical method, the ideal residual stress field distribution of GH3039 superalloy can be obtained by adjusting the laser processing parameters to meet the service requirements of aerospace components.

## Figures and Tables

**Table 1:** The chemical compositions of GH3039 superalloy (wt %).

C	Cr	Al	Ti	Mo	Nb	Fe	Si	S	Mn	P	Ni
0.051	21.00	0.57	0.59	2.10	1.11	3.00	0.80	0.012	0.40	0.020	Bal.

**Table 2:** Surface residual stress test results of 2 mm thick LSP treated GH3039 plate.

No.	1	2	3	4	5	6	7
Val.	−319.3	−326.5	−324.1	−309.5	−318.1	−324.8	−311.2

## Data Availability

The original contributions presented in this study have been included in the article. Further inquiries can be directed to the corresponding authors.
